# Genetic material from buffalo and cattle: crucial importance in the formalization of bilateral trade between India and Brazil

**DOI:** 10.1590/1984-3143-AR2020-0031

**Published:** 2020-11-10

**Authors:** Eduardo Bastianetto, Denise Aparecida Andrade de Oliveira, Concepta McManus, Dalci de Jesus Bagolin, Rômulo Cerqueira Leite, Cristiano Barros de Melo

**Affiliations:** 1 Escola de Veterinária, Universidade Federal de Minas Gerais, Belo Horizonte, MG, Brasil; 2 Programa de Pós-graduação em Ciências Animais, Universidade de Brasília, Brasília, DF, Brasil; 3 Embassy of Brazil, Ministry of Agriculture, Livestock and Food Supply, New Delhi, India

**Keywords:** animal health, Brazilian buffalo populations, genetic composition, international health security

## Abstract

The trade in live animals between India and Brazil dates from the late nineteenth century when European travellers traded animals of Indian origin for display in zoos. Considering the origin of coffee and sugar cane, as well as the expertise related to mineral evaluation, we need to consider that India was involved in important economic cycles of Brazil, even indirectly. This virtuous flow of trade has been maintained and intensified throughout modern history, especially after these two nations gained political independence from their colonisers, thereby becoming independent in mercantile affairs. This paper addresses the main points related to the use of animals of Indian origin in Brazil. We revisit some of the historical aspects of the process of colonisation of Brazil, as well as the importation of animals from India. The restrictions imposed on this process due to the occurrence of diseases in cattle and buffalo in India will be examined. At the end of the text, emphasis will be given to the risks of introducing exotic diseases into Brazil.

## Introduction

### The Brazil-India relationship

Brazil was the first country in Latin America to establish diplomatic relations with independent India (1948). Several cooperation agreements were signed in several areas, including agriculture, biotechnology, environment, biofuels, and cosmic space. Additionally, Brazil facilitated an unprecedented convergence between India and the economic bloc called the “Southern Common Market” (MERCOSUR) (Vazquez, 2019; Viswanathan, 2019). In 2009, the MERCOSUR-India Preferential Trade Agreement came into force, becoming the first instrument of its kind signed by the bloc outside the region (Nunes, 2019). The strategic partnership established between Brazil and India has expanded since 2006, with both countries cooperating closely within economic blocs from countries such as BRICS, IBSA, G4, G20, and BASIC, as well as the broader multilateral context of the United Nations. Bilateral dialogue and cooperation have also increased. They should gain more density and autonomy, as there is still great potential to be explored in the context of India-Brazil relations. Indo-Brazilian collaboration can be expanded in the areas of science, technology and innovation, trade and investment, defense, sustainable development and combating poverty, governance, multilateral cooperation, and civil society exchanges (Nunes, 2019). In addition to bilateral cooperation, India and Brazil, together with South Africa, formed the IBSA trilateral forum and later worked together on the BRICS agreement. At the United Nations, the two countries acted proactively to reform the United Nations Security Council and aspired to a seat of permanent members of the body. Bilateral trade between Brazil and India is also reflected in flora and fauna and in food and folk traditions, on both sides. Indian plants (mango, coconut, sugar cane, and jackfruit) were introduced in Brazil, while others (cashew and cassava) were exported from Brazil to India (Hamilton and Zug, 1998; Ribeiro et al., 2013). Similar cultural activities in both countries (Boi Bumbá in Northern Brazil and Poikham Kudhrai in Southern India) point to the strong hidden currents of cultural and popular exchanges that have taken place between both countries (Vazquez, 2019; Viswanathan, 2019).

Brazil is an agricultural superpower. It is not only self-sufficient in food security but it exports its surplus and is a global player in agribusiness. The country is the world's largest exporter of beef, chicken, sugar, soy, orange juice, and coffee, in addition to being an important exporter of corn, cotton, tobacco, bananas, pork, and ethanol. It is predicted that Agricultural Production from Brazil will increase in coming years (USDA, 2020) and its exports feed hundreds of millions of people worldwide. With scientific progress, Brazil has inserted millions of hectares of arid land for cultivation (Pereira et al., 2012). However, it is still part of the developing world with poverty problems, among other issues. Brazil is not dependent on any particular country or region for trade (Baumann, 2010), as its foreign trade is diversified between the European Union, USA, China, and MERCOSUR. Additionally, Brazil still has enormous challenges, such as inequality, insecurity, drug trafficking, corruption, infrastructure, healthcare, and education (Viswanathan, 2019). During the 70 years of diplomatic relations between Brazil and India (Vazquez, 2019), 55 bilateral trade agreements were signed, in addition to the creation of a fund for India, Brazil, and South Africa (IBSA) to combat poverty, as well as others areas. Four areas represent more than 40% of these agreements: education, technical cooperation, culture, and immigration. These areas offer possibilities to deepen Brazil-India relations. These cooperation agreements have become increasingly reflected in the exchange of professionals between the two countries and in the mobility of students in higher education, especially Brazilians to India. The presence of Indian laboratories in the Brazilian pharmaceutical market is also significant, surpassing the participation of European laboratories in particular niches (Franculino and Gomes, 2017). Thus, there is strong cooperation between Brazil and India in education, science, and industry. This dynamic depends on public and private sectors relevant to bilateral win-win cooperation (Uebel, 2019; Vazquez, 2019).

India's most challenging problems include the provision of food, education, healthcare, and infrastructure to its additional 15 million inhabitants each year. India has several day-to-day issues of conflict between communities and other problems arising from the vast diversity of the country, which has 22 official languages but some 1500 languages and dialects (Laitin, 1989). In this sense, India sees Brazil as an ideal partner for global strategy. The two countries have a shared worldview and aspirations and face similar challenges. Over the years, India and Brazil have built a working relationship in many global forums and multilateral negotiations. Brazil is the largest economy in Latin America and India is its largest trading partner in the region, with bilateral trade of US $8.6 billion in 2017-2018 (Vazquez, 2019; Viswanathan, 2019).

Currently, both countries also have to deal with environmental issues and economic inequality, as a large number of people still live below the poverty line. Both countries depend on the efficient use of energy sources to deal with economic and environmental challenges, and for that they have presented essential projects related to renewable energies. Brazil created the Biofuels Platform to leverage biofuel markets (Wilkinson and Herrera, 2010). In addition to India, this Platform has been ratified by 20 countries. India, for its part, launched the International Solar Alliance to promote solar energy in developing countries. The agreement was ratified by 120 countries, including Brazil. In 2015, the ministers of foreign affairs of Brazil and India expressed interest in strengthening cooperation in the area of renewable energy and in this way, all these facts showed that the two countries are converging on the development of renewable energy (Mousinho, 2019; Vazquez, 2019).

Brazil and India also have common interests related to combating poverty and corruption (Studnicka, 2010; Ravallion, 2011), strengthening and simplification of the tax system and fiscal federalism, expansion and improvement of banking system regulations, inclusion of the poorest in the credit system, improvement and rationalisation of payment systems, attracting foreign investments, and increased participation in international trade. In this sense, cooperation within successful experiences for good governance can be essential and enhance progress for both countries, within the scope of the international order (Carvalho et al., 2019; Vazquez, 2019).

Both countries experience the same problems in terms of gangs and terrorism (Sullivan, 2002). The governments of the two countries strive to combat the financing of criminals in India, especially terrorism and corruption in Brazil. Although the connections between Brasilia and New Delhi have been described as a “strategic partnership” since 2006 that has continued to strengthen, as pointed out by the Annual Report of the Ministry of Foreign Affairs of India in 2018, there are still possibilities to be explored (Carvalho et al., 2019; Vazquez, 2019).

Biotechnology is another area that may be important in India-Brazil cooperation (Pray, 2001). Biotechnology integrates concepts from biology, chemistry, engineering, and information technology to extract products and services from the living system to be applied in the optimisation of human health, animal health, agriculture, and use of environmental resources and energy sources. The biotechnology sector in India is ranked among the top 12 biotechnology centers in the world and has around 800 fully functioning companies. Biopharmaceuticals are the main branch of the Indian biotechnology industry (Franculino and Gomes, 2017), responsible for 62% of the sector's revenue, mainly due to the country's significant participation in the global generic drug market. In addition, products for human health, biological services (18%), and bio-agricultural products (15%) constitute the main potential of Indian industry in the area of ​​life sciences. In this sense, the potential for increasing Brazilian productive capacity in biotechnology is significant. In Brazil, the main niche of products in the biotechnology market is the human health sector, which accounts for 39.7% of companies, followed by environment and bioenergy (14.8%), animal health (14.3%), and agriculture (9.7%). Brazil is also a world leader in the production of bioplastics and biofuels from ethanol, thus there is a complementarity between the two countries (Nascimento, 2019). This bilateral relationship can be initiated by the similarity of objectives and support strategies to the sector, considering research related to seeds, human health, manufacturing of vaccines, and recombinant gene therapy, in addition to the global market for industrial enzymes. The latter aims to convert polluting chemical methods into sustainable production processes (Nascimento, 2019; Vazquez, 2019).

Brazil and India can also cooperate bilaterally on topics, such as the preservation of biodiversity, exploitation of indigenous knowledge for commercial purposes that has a far-reaching impact on general equity, and scientific development and innovation, as the abuse of biological resources and knowledge associated by companies produces deleterious effects at the macro level (Eimer, 2014). India and Brazil have the highest biodiversity in the world. They can cooperate in protecting the rights of their indigenous peoples, genetic resources, and the knowledge associated with them, since biopiracy is rampant. These acts are categorized in the patenting of inventions that were developed using biological resources or indigenous knowledge extracted illegally or without the necessary authorization from the appropriate authorities (Oyewunmi, 2013). They tend to not give any credit to the local communities that may have helped in this development, defined as patent-based biopiracy, intellectual property rights (IPR) not related to bio-based technology patents but developed in the same way, and finally, the unauthorized extraction of biological resources and indigenous knowledge without proper sharing of benefits or attribution of credit to local communities is known as “embezzlement”. Thus, it is also essential to advance their legal framework for the protection of biodiversity in both countries through cooperation (Vazquez 2019; Tripathy, 2019).

Brazil and India also play an essential role in the United Nations and have a history of participating in peacekeeping operations. The knowledge accumulated in Peacekeeping Operations (PKOs) can be used to promote relations between the two countries, as well as to help the UN strengthen peacekeeping and peacebuilding. Currently, PKOs are tasked with providing security to assist in civil reconstruction, demobilize armed groups and government forces, collect weapons, assist refugees and internally displaced persons, organize elections, monitor human rights, support national dialogue and reconciliation, develop institutions and administrative capacity, and restore the rule of law, among others (Stuenkel, 2010). These operations to manage or resolve conflicts are related to the authorization of the United Nations Security Council (UNSC) to use force to protect civilians under imminent threat of physical violence (Alden and Vieira, 2005). Diplomatically, Brazil and India share the view that force should be part of an extensive peace process and used as a last resort when necessary to provide security for civilians under threat of physical violence. Thus, Brazil and India have the potential to build “new” perspectives on maintaining and building peace, in addition to being able to advance bilateral relations by sharing experiences and knowledge, working together at the UN, and establishing common positions in the political, strategic, and operational military (Vazquez, 2019; Aguilar, 2019).

Still, considering bilateral cooperation in the areas of defense and security, India has been the largest arms importer in the world for almost a decade and practically does not buy weapons and military equipment from Brazil. Thus, the two countries have not yet identified a mutual interest in arms transfer, especially concerning Indian imports into Brazil (Brauer and Dunne, 2011). Bilateral cooperation may, in addition to involving arms transfer, involve mutual collaboration in various areas related to defense, such as research, supply, purchasing, and logistical support, in addition to complementary activities, such as educational exchange in military schools, joint training for maintenance operation peace, military participation in simulations and training programs, mutual involvement in strategic projects, partnerships related to technology, sharing of best practices, and high-level visits, among others (Brito et al., 2019; Vazquez, 2019).

## Cattle and buffalo herd formation in Brazil and its impact on the current genetic composition of purebred animal populations

The trade in live animals between India and Brazil dates back to the late nineteenth century (Santana et al., 2016) when European traders traded animals of Indian origin for display in zoos. Before this, animals hitherto existing on Brazilian soil were all of European origin, brought for the establishment of productive practices at the time of colonization (Mariante et al., 2009). Cattle and buffalo populations began to grow at a faster rate in Brazilian territory with the transfer of individuals from India to Brazil, followed by their uptake with private farmers, due to the perception of greater productive capacity of animals of Indian origin in Brazilian territory. Between 1870 and 1962, only 2465 cattle animals (*Bos taurus indicus*) were imported from India. The Brazilian population of *Bos taurus indicus* was mostly produced by “grading up”, mating *Bos taurus ibericus* cows brought to America by Portuguese and Spanish colonists to *Bos taurus indicus* bulls, imported from India (McManus et al., 2009). Among the cattle breeds present in Brazil, the Nellore breed has the largest number of individuals, both with the standard (horned) and polled phenotype, followed, in order of magnitude, by the Guzerat, Gir, and Indubrasil breeds (Ferraz and Felício, 2010). The formation of new genetic composite breeds using pure bred *Bos taurus indicus* individuals has become quite common, especially for the Gir and Guzerat breeds, with Ongole (Nellore) animals, drastically reducing the number of purebred individuals. Because of this mating process among the Zebu breeds in Brazil, the Tabapuã breed (Santiago, 1985) was created.

The genealogical record of cattle breeds imported from India began in 1938, starting the database of genetic origin, together with the collection of productive indices (Josahkian, 2000). This database was used in a recent study that evaluated the genetic composition of cattle of Indian origin using modern assessment tools, such as molecular markers (microsatellites and SNPs), and high consanguinity was demonstrated (Tapio et al., 2006). Santana et al. (2016) evaluated data from the national pedigree archive of the seven Brazilian Zebu breeds (Brahman, Gir, Guzerat, Indubrasil, Nellore, Sindi, and Tabapuã), including all animals registered (12,290,243) (born) between 1938 and 2012. Almost all breeds studied are expanding without maintaining genetic diversity. These authors reported the most common problem as the presence of bottlenecks in the pedigree, which occurs mainly due to the intensive use of few animals as parents and the high degree of subdivision in the population. Campos et al. (2017) demonstrated that the Gir breed had low racial purity at the genomic level due to its very heterogeneous mixing pattern.

Regarding the variation in the constitution of the genetic composition of cattle raised in the Brazilian territory, Brasil et al. (2013) carried out a study that sought to evaluate the evolutionary relationship between the breeds (phylogenetic relationship) with levels of genetic diversity and the patterns of mixtures taurine/zebu, among nine commercial breeds (Brahman, Gir, Girolando, Guzerat, Dutch, Jersey, Nellore, Senepol, and Tabapuã) reared in Brazil. A total of 2,965 animals were analyzed using 11 microsatellites. The main objective of the study was to understand the genetic diversity of these populations, which is of fundamental importance in management, breeding, and conservation programs. The data also showed a higher degree of differentiation (distance) within the Taurine breeds compared to within Zebu breeds in Brazil. In other words, Taurine breeds have less gene exchange than Zebu breeds, presenting themselves as more “purebred” than Zebu breeds (Brasil et al., 2013).

This pattern probably reflects the way in which these two groups were introduced and are managed in the country. No breed classification was used during the first decades of introduction of Zebu cattle and all animals from India were generically called Zebu. The racial pattern of Zebu breeds was described and implemented in 1938 (Santiago, 1985; Josahkian, 2000; Brasil et al., 2013). Thus, genetically pure herds, directly descended from imported animals and without introgression (introduction) of Taurine genes, are very rare today. This last hypothesis was corroborated by the mixing pattern observed for Zebu breeds. This pattern has also been reported for several other Latin American breeds (Mariante et al., 1999; Egito et al., 2007; Delgado et al., 2012; Brasil et al., 2013).

There is significant diversity shared between breeds developed in the Americas over the past 100 years. Despite their relatively recent introduction, these breeds comprise distinct subpopulations and thus, act as an important source of diversity in the tropics. Despite the significant introgression observed, especially among the Zebu breeds, it was possible to distinguish which breed an individual animal belongs to, based on the genetic data described, in up to 90% of cases (Egito et al., 2007; Brasil et al., 2013).

The Senepol breed is a relatively isolated group among Taurine populations, since it was formed by crossing N'Dama races from Africa with the European Red Poll breed in an isolated environment on the Caribbean island of St. Croix. The Girolando breed was between European Taurine and Indian Zebu breeds rather than being observed on a separate branch. The Girolando was derived from the 5/8 Holstein cross between Holstein and Gir (Brasil et al., 2013).

European cattle were introduced in Brazil approximately 500 years ago. However, large-scale commercial production was established only after the import of Zebu breeds from India in the late 19th and early 20th centuries (Mariante and Egito, 2002). The Zebu breeds easily adapted to tropical conditions in Brazil. They quickly became prominent in the formation of herds, contributing to the development of new breeds in the Americas, such as Tabapuã, Indubrasil, and Girolando (Ferraz and Felício, 2010).

Other commercial breeds reared in Brazil, such as Brahman and Senepol, were also formed in the last century but in other countries on the American continent. The majority of the Brazilian cattle herd is thus composed of breeds of Zebu origin (Dani et al., 2008). The participation of bovine breeds of Indian origin (*Bos taurus indicus*) in the formation of the genetic base of the bovine herd in Brazil, in particular, the Nellore, Gir, and Guzerat breeds, is one of the reasons for the success of productivity observed in some herds. Even when using animals of Indian origin for the formation of composite breeds, formed by crossing *Bos taurus indicus* x *Bos taurus taurus*, as observed in the Girolando breed, the adaptation of crossbred animals to the production environment in Brazil may stem from the phenotypic manifestation of genes from Zebu in the genetic composite (Brasil et al., 2013). Nevertheless, Brazilian local breeds have also been seen to confer this adaptability (McManus et al., 2009).

As for the bubaline species (*Bubalus bubalis*), animals from the Murrah, Jafarabadi, Mediterranean, and Carabao breeds were introduced in Brazil in a smaller amount but assumed importance in the Northern Region of Brazil due to their greater ability to survive under extreme conditions (Camarao et al., 2004; Andrade and Garcia, 2005). In some situations, the higher productivity of buffaloes occurs due to their greater ability to survive in extreme swamp or drought conditions, in the presence of toxic plants and forages of low nutritional value, producing in this scenario, traction and food, as well as other raw materials for the sustenance of humans (Vale et al., 2013). Currently, not only due to the limited production capacity of other species of ruminants but also for economic reasons, the population growth of the buffalo species in Brazil deserves attention, which was more than 30% in the period from 1996 to 2018, totalling more than 1,390,066 animals (Andrade and Garcia, 2005; IBGE, 2018).

The reduction in the genetic variability of these populations, in addition to the common origin, is a further point of convergence between the purebred individuals from tropical herds of bovine and buffalo species in Brazil (Marcondes et al., 2014). Since the last importation of animals from India in 1962, both in Brazil and India, breeding animal selection programs have been developed, aiming at genetic improvement to increase milk productivity (Marques et al., 2019), as well as meat quality (Cassiano et al., 2004; Canozzi et al., 2016).

In Brazil in 1960, the Brazilian Association of Buffalo Breeders (ABCB, 2020), a private entity, was created to disseminate and collaborate for technical support related to the creation of buffaloes in Brazil. ABCB also has the function of maintaining a mating database for controlling the genealogy of animals, according to guidelines imposed by MAPA. At the regional level, actions are also taken to improve the genetics of the buffalo population in each location through regional breeders' associations or programs supported by a state agency, such as Promebull (Silva et al., 2016; EMBRAPA, 2018). In India, through the Cattle and Buffalo Development program, various central and centrally sponsored schemes are being implemented for the genetic improvement of cattle and buffalo (Sreenivas, 2013) to enhance the *per capita* consumption of milk through increased milk production. Efforts are also being made to protect and preserve indigenous cattle and buffalo in their native tract, which are facing the threat of extinction. The elite animals are selected and registered based on their performance for the production of superior pedigree bulls, bull mothers, frozen semen, and frozen embryos for future breeding improvements (India, 2011).

According to ABCB, and also present in the small amount of literature about the introduction of the buffalo species in the national territory on the arrival of buffalo in Brazil (Albuquerque et al., 2006; ABCB, 2018), these animals came in small lots at different times. The first group was of Carabao or Rosilhos buffalo in the Amazon in the 1890s. In summary, the importation of individuals of the buffalo species of Asian origin began in 1895, followed by occasional episodes, usually accompanying cattle imports (Andrade and Garcia, 2005). There were few episodes of direct and exclusive importation of buffalo for the use of this species in production systems in Brazil (Andrade and Garcia, 2005). Similar to that observed in cattle species, animals were initially mated without observing or defining a breed pattern. Most recently, in 1989, Mediterranean animals were imported from Italy to the states of Sao Paulo, Rio Grande do Sul, and Bahia (ABCB, 2018), just as semen from Murrah and Mediterranean breeds were imported from Italy and Bulgaria. Imports of semen from Mediterranean animals of Italian origin have been carried out in recent years to meet the urgent demand of buffalo breeders who could not find options in the national market for the mating of females, regardless of the predominant breed on the farm (Ribeiro and Vale, 2007). The Brazilian Association of Artificial Insemination reports low production of buffalo semen in Brazil, which totalled 11,727 doses of Murrah semen in the period from 2014 and 2017 and 784 from Jafarabadi in 2016 (ASBIA, 2017).

To better understand the current composition of the national buffalo herd, Vieira (2014) characterised this population using microsatellites and found low heterozygosity deficit (Fis = 0.077), with intermediate genetic structure in the populations evaluated. This was attributed to the high index of inbreeding present in the evaluated properties and the poor selection of bulls used in the mating and the crossing between river (2n = 50 chromosomes) and Swamp (2n = 48 chromosomes) buffalo, upon the introduction of buffaloes in Brazil. Over the years, breeders and breeding stock were selected without due concern for the mating schedule, which favoured, even unintentionally, the occurrence of inbreeding. As a consequence, high levels of inbreeding occurred, which justifies the introduction of new germlines in the herds of River buffaloes, so that the genetic variability can be increased (Marcondes et al., 2014) and thus, decreasing the degree of inbreeding existing and thereby its impact on the productivity of herds (Vieira, 2014).

Considering the need to import cattle and buffalo genetic material from the Asian continent, it is also essential to check the phenotypic and genotypic quality of donor animals. This is necessary so that the imported genetic material can genuinely contribute to the improvement of the Brazilian herd and, thus, be inserted in the official genealogical control system in Brazil.

In modern production systems, where animals of the same breed tend to be selected for the same phenotypic characteristics, a reduction in reproductive capacity has been observed (Taberlet et al., 2008), which may be a consequence of the high inbreeding levels of purebred herds of Zebu animals raised in Brazil. Cattle of Asian origin (*Bos taurus indicus*) are considered to be more adapted to production systems and climatic conditions in tropical regions, such as Brazil, than those of European origin, with the exception of some locally adapted breeds (McManus et al., 2009). This is due to hair and skin characteristics of these animals, as well as digestive and immune systems of breeds that underwent a long process of selection and adaptation for greater tolerance to the most common diseases in the tropical environment (McManus et al., 2020). To preserve populations, genetic diversity within the appropriate breed needs to be maintained (McManus et al., 2010) to ensure long-term sustainable exploitation of livestock, especially in light of anticipated climate changes that include rising average temperatures (Romanini et al., 2008; Scholtz et al., 2010). The above considerations confirm the need for the introduction of new lines of high genetic value in the cattle and buffalo population of Brazil.

One could imagine that buffalo breeding would be solely directed to fill the so-called cattle breeding gaps, however, it has been seen that, in those areas where breeders could successfully organize industrial-agricultural chains, either for meat or milk and their related products, there has been a significant expansion in herd numbers. In Brazil, buffalo breeding has shown to be an important alternative, not only in farms of higher technological level, but also on small farms, where it has become a key factor for increasing average income, as well as keeping the labor force in country areas (Bernardes, 2007). Under inadequate environmental conditions for cattle breeding, such as Marajo Island, water buffalo maintain an essential role in the elevation of the social and economic level of small communities (Gill, 1984). In Brazil, especially in the Amazon region, they can be of great importance for milk production in small and medium rural properties. Still, it is necessary to understand the relationships between productive variables (such as milk production) and environmental variables (climate and nutrition, mainly) (Lourenço-Junior et al., 1999).

## Imminent health risks of clandestine importation of cattle and buffalo biological material of Indian origin without supervision of agricultural authorities

Concern about the ban on the importation of live animals from India to Brazil after 1962 is still justified today, especially considering the presence of exotic infectious agents in India that can be brought to the national territory. Diseases and infectious agents may enter countries and regions through irregular transit of live animals and the use of biological material of animal origin in assisted reproduction processes, such as artificial insemination or embryos. In the case of the introduction of infectious diseases, there are many sanitary barriers in international trade that imply high investments for their control and severe losses in outbreaks (Garcia et al., 2015). In the case of the introduction of exotic microorganisms or genetically different strains from those present in Brazil, the loss to animal health of the herds in Brazil, as well as the commercial market, is incalculable.

Often, ranchers or even technicians without veterinary medicine training, mistakenly believe that the freezing process and the maintenance of biological material in liquid nitrogen at a temperature of -196°C is sufficient to eliminate the risk of transmission of infectious agents (Carvalho et al., 2007). High-risk infectious agents for Brazilian livestock, mainly those that have never been detected in Brazil, can be introduced, such as for example, the contagious caprine pleuropneumonia, foot and mouth disease, and peste des petits ruminants, according to the World Organization for Animal Health (OIE) (OIE, 2012).

As for foot and mouth disease (FMD), three serotypes of the FMD virus (O, A, and Asia1) were prevalent in India and serotype O was responsible for 80% of confirmed outbreaks/cases, whereas Asia1 and A caused 12% and 8%, respectively (Subramaniam et al., 2013). Serotype C has not been encountered in India since 1995 and the reason for its abrupt disappearance is not known. In India, continuous antigenic divergence in serotype A resulted in changes in the vaccine strain in 2009. The authors state that, to date, all genetic diversity within the serotypes is well tolerated by the vaccine strains. Currently, a trivalent (O, A, and Asia1) vaccine is used in the vaccination program. Unrestricted animal movements in the country play a significant role in the spread of FMD. In India, despite the Indian government's efforts to contain the disease, a total of 2,669 outbreaks/cases were recorded between April 2006 and March 2011 and have involved cattle (94.5%), buffaloes (3.3%), pigs (1.21%), sheep (0.115%), and 1.4% of the samples were from semi-domesticated and wild animals including Mithun, Yak, Nilgai, and Gaur (Pattnaik et al., 2012).

Ranaweera et al. (2019) sequenced the VP1 genomic region of FMD viral isolates belonging to serotype C from Sri Lanka and other South Asian countries. All published VPI sequences of serotype C and most of the published VP1 sequences for lineage ME-SA/Ind2001d of serotype O from Sri Lanka, India, and other South Asian countries were retrieved. The datasets of serotype C and serotype O were analysed separately using Bayesian, maximum likelihood, and phylogenetic networking methods to infer the transboundary movements and evolutionary aspects of the FMD virus (FMDV) incursions in Sri Lanka. A model-based approach was used to detect any possible recombination events of FMDV incursions. The results revealed that invasions of the topotype Asia of serotype C and the lineage ME-SA/Ind-2001d have a similar pattern of transboundary movement and evolution.

In 2005, the introduction of FMD to Brazil by the border of the state of Mato Grosso do Sul occurred, due to the irregular transit of cattle from Paraguay, with outbreaks extending until 2006, which caused significant losses in Brazil (Garcia et al., 2015). Immediately following the declaration of a focal point, a list was published by the Ministry of Agriculture, Livestock and Supply (MAPA) that reached 58 countries. These imposed official restrictions on Brazil, representing 86.74% of the international consumption of Brazilian meat. Confirmation of the first outbreak of FMD in 2005 was followed by embargoes on Brazilian meat, which had a substantial impact on the country and the indicator “ESALQ /BM&F beef with cash payment” decreased by 8%, and prices behaved atypically with devaluations. Thus, it took eleven months for the market to recover its value from the fall of the previous year (CEPEA, 2005; Garcia et al., 2015).

Brazil reported the last outbreak of FMD in 2006 (Carvalho et al., 2014) and the Brazilian government made a significant effort to eradicate this disease in its territory (Brasil, 2019), which helped to keep the country as one of the largest meat exporters in the world. Brazil received recognition as a FMD-free zone with vaccination (OIE, 2019b) from the OIE in May 2018. Currently Brazil is preparing to completely withdraw FMD vaccination (Brasil, 2019). Serotype Asia-1 is exotic in Brazil and the serotypes A and O, prevalent in India, also may not be recognized by vaccines produced in Brazil, as they are probably of different viral subtypes than those prevalent in Brazilian vaccines. Brazilian vaccines were trivalent (serotypes A, O, and C) (Brasil, 2019), however, since 2019, bivalent vaccines (O and A) have been applied in Brazilian herds, due to the conclusion of the non-existence of serotype C in South America. Thus, Brazilian vaccines do not provide protection against Asia-1 serotype, which increases concern and makes FMD a disease that needs close surveillance in the import processes of animals and their by-products from countries where FMD is present.

Around 1920, Rinderpest came into Brazil from cattle imported from India by sea, after a stopover in Antwerp Port, Belgium. The imported Zebu herd arrived infected at the Port of Santos in São Paulo, which caused significant damage to the production system at the time. This dire situation led to the creation of the OIE itself in 1924, based in Paris (Magalhães, 1922; OIE, 2020).

Diseases and infectious agents may also enter countries and regions through the irregular transit of animal products/by-products, such as milk, cheese, meat, sausages etc., as shown in studies at Brazilian airports (GRU - Guarulhos International Airport/São Paulo in São Paulo and GIG - RIOgaleão - Tom Jobim International Airport in Rio de Janeiro) to show these risks (de Melo et al., 2014 a , b, 2015, 2018). The illegal entry of animal products brought through international passenger baggage at Brasília–Presidente Juscelino Kubitschek International Airport (AIB-SBBR) in Brasilia was studied by de de Melo et al. (2016). In a terrestrial modal, Eidt et al. (2015) studied international agricultural surveillance at the borders of Brazil with Peru, Bolivia, and Venezuela, reinforcing the need to expand the prevention of the entry of live animals, their products/by-products, and genetic multiplication material, among others, illegal by these borders.

Brazil has also studied and monitored the transit of live cattle exports, mainly to Lebanon, Angola, Egypt, and Venezuela, mainly for slaughter by sea from the state of Pará (Sá et al., 2018). Tools for this animal traffic monitoring have been proposed, such as the use of satellite images for georeferencing farms (Carvalho et al., 2012). Works like these generate essential information that enables the reduction of health risks and better prediction of potential impacts on future economic scenarios, which may affect partners on global issues.

Embryo marketing is less dangerous and, despite the need for growth in production rates and renewal of the genetic status of herds, the risk has to be well evaluated (Carvalho et al., 2007). The transfer of biological material through embryos is the safest option, primarily when embryos are produced and stored in companies that meet OIE health standards. According to the International Embryo Transfer Society - IETS (Stringfellow and Seidel, 1998), embryo transfer can be an effective and safe means of preventing the transference of many pathogens in the international trade in genetic material. This is especially notable when appropriate collection techniques are used, as well as safe handling and transfer and the identification of embryos is adequate to guarantee the relationship between official certification and commercialized embryos.

Considering the sanitary situation of cattle and buffalo farming in India and analyzing the risk of transmission of these infectious agents, FMD presents an insignificant risk of transmission by embryos but a significant possibility of transmission by semen. Regarding *Contagious caprine pleuropneumonia*, this infectious agent, transmitted by semen, has significant risk and is likely to be transmitted by embryos (Carvalho et al., 2007).

Therefore, there is a need to reduce or mitigate the risk of introducing infectious agents and diseases through the formalization and establishment of internationally accepted protocols, led by MAPA and the Ministry of Foreign Affairs, together with their official counterparts in India.

## Current situation of trade relations of biological material for animal reproduction between Brazil and India

Understanding the importance of the bilateral restructuring of trade in genetic material and livestock production processes that guarantee the sustainability of production, the Government of India is analyzing models of zoosanitary certificates for the importation of biological material from different sources presented by the Government of Brazil. In this regard, the following sanitary and zoosanitary certificates from Brazil are in force and approved by the Government of India, including imports of meat and chicken (2008), pets (2013; dogs), cattle semen (2015), fertile eggs, day-old chicks (2016), pets (2017; cats), *in vivo* cattle embryos (2018), pigs and live cattle for breeding (2018), and SPF eggs (2018), while pig meat and *in vitro* cattle embryos are still being analyzed (Brasil, 2013, 2017, 2018, 2020).

In the specific case of the importation of buffalo and bovine genetic material, there are established standards worldwide. There are certification standards for the trade of genetic material of various species detailed in the OIE Terrestrial Animal Health Code, which is a recognised reference by the World Trade Organization (WTO) (OIE, 2019a). Although OIE standards are routinely used for trade between countries, they are only guidelines and countries can establish more or less restrictive import standards and measures according to the risks identified in the country of origin or the product to be imported (Stringfellow and Seidel, 1998; Carvalho et al., 2007).

In 2018, the Brazilian government initiated a similar action by forwarding, through its embassy in New Delhi, to the Government of India, a document requesting analysis of Brazil's requirements for the importation of buffalo semen and embryos from India (Bastianetto, 2018). Following this initial request, other documents were presented to reinforce the need for regularization of the process of importation of genetic material from India by Brazil, in particular for the use of genetic material in buffalo breeding for regional development programs aimed at reducing local poverty and improving child nutrition in schools.

The formalization of trade in genetic material between these two countries is essential to favor food production processes in Brazil, in which cattle and bubaline species are of direct importance, as well as intensive international surveillance aimed at curbing irregular trade in genetic material to ensure a profitable and secure business for both parties. As stated, the creation of a bilateral committee that can follow up work on buffalo breeding can be an important step in making this process successful.

## Final considerations

As the central theme under discussion, the proper presentation of health risks presented by the reactivation of the commercial activities of genetic material between the two countries is important, especially in the in case of non-compliance with good production and diagnostic practices, under already existing sanitary protocols. This mutual responsibility of Brazil and India for the health and security of cattle and buffalo herds should be a coordinated action to reactivate trade in this type of material to promote food productivity and security.

Countries should also be responsible for surveillance of their borders to curb illegal trade in cattle and buffalo genetic material. To this end, legal trade mechanisms need to be established with the permanent involvement of surveillance and disease diagnosis systems in each country. In the event of outbreaks of an exotic disease, whether in Brazil or India, from clandestinely imported material, a severe crisis in food production and foodstuffs of animal origin can occur, affecting the economy and national credibility with other countries with which Brazil and India maintain commercial relations. Therefore, these rules need to be clear and followed by all parties involved.
